# Radiofrequency vs. Microwave Ablation in Osteoid Osteoma: Comparative Outcomes and Prognostic Factors

**DOI:** 10.3390/jcm14217814

**Published:** 2025-11-03

**Authors:** Ismail Karluka, Mustafa Mazıcan, Cagatay Andic, Cagatay Bolgen, Salih Beyaz, Necmettin Turgut, Alaaddin Levent Özgözen, Hakkı Can Ölke

**Affiliations:** 1 Department of Interventional Radiology, Başkent University Dr. Turgut Noyan Application and Research Center, Adana 01250, Turkey; m_mazican@yahoo.com (M.M.); cagatayandic@gmail.com (C.A.); 2 Department of Interventional Radiology, Medline Hospital, Adana 01170, Turkey; drcagataybolgen@gmail.com; 3 Department of Orthopedics and Traumatology, Başkent University Dr. Turgut Noyan Application and Research Center, Adana 01250, Turkey; sbeyaz@baskent.edu.tr (S.B.); drnecmettinturgut@hotmail.com (N.T.); leventozgozen@yahoo.com (A.L.Ö.); drhcanolke@gmail.com (H.C.Ö.)

**Keywords:** osteoid osteoma, radiofrequency ablation, microwave ablation, image-guided ablation, clinical success, recurrence, prognostic factors, interventional radiology

## Abstract

**Background**: Osteoid osteoma (OO) is a benign osteogenic tumor that causes severe pain despite its small size. Minimally invasive image-guided thermal ablation has replaced surgery as the treatment of choice. While radiofrequency ablation (RFA) is considered the gold standard, microwave ablation (MWA) offers faster and more homogeneous heating, though comparative evidence remains limited. **Methods**: We retrospectively analyzed 53 patients with OO treated with RFA (n = 27) or MWA (n = 26) between 2014 and 2023. All procedures were CT-guided. Technical success, clinical success, recurrence, complications, and prognostic factors—including the nidus diameter and eccentricity index—were evaluated over a minimum 24-month follow-up period. **Results**: Technical success was achieved in all cases. Overall clinical success was 94.3% (96.2% MWA vs. 92.6% RFA, *p* = 1.000). Two recurrences (4%) occurred, unrelated to device type. One major complication (1.9%, third-degree skin burn after MWA) was noted. Median nidus diameter was 7 mm; lesions ≥10 mm were significantly linked to failure (*p* = 0.009). Logistic regression identified nidus size as the strongest outcome predictor, with the eccentricity index showing a borderline effect. **Conclusions**: Both RFA and MWA are safe and effective for OO, with comparable outcomes and low recurrence rates. Treatment selection should prioritize lesion-specific factors—particularly nidus size ≥ 10 mm and geometry—rather than device type. Lesion size (≥10 mm) and geometry—not ablation modality—were the principal determinants of treatment success. Individualized modality selection based on these features may optimize outcomes.

## 1. Introduction

Osteoid osteoma (OO) is a benign osteogenic tumor that predominantly affects young individuals. Despite its small size (<2 cm), it produces clinically significant symptoms and accounts for 10–14% of benign bone tumors. The condition is characteristically associated with prostaglandin-mediated nocturnal pain that responds rapidly to nonsteroidal anti-inflammatory drugs [1,2]. OO most frequently arises in the meta-diaphyseal regions of the femur and tibia, although axial and small bones may also be involved. Surgical excision, once considered the standard of care, has largely been supplanted by minimally invasive, image-guided thermal ablation techniques due to lower morbidity and shorter recovery [1,2]. Among these, radiofrequency ablation (RFA) remains the most established approach [3], while microwave ablation (MWA) has gained increasing interest due to its ability to achieve faster and more homogeneous heating.

RFA is currently regarded as the reference standard for the minimally invasive management of osteoid osteoma, with large series reporting technical success rates approaching 100% and clinical success exceeding 90% on long-term follow-up. Reported complication rates are low (approximately 2–3%), and recurrences can typically be managed effectively with repeat ablation [4]. These low complication rates and the feasibility of retreatment have been consistently documented across cohorts [4]. Initial reports and small patient series have demonstrated clinical success rates ranging from 92% to 100%, with no significant differences in recurrence or complication rates compared to RFA [4]. Taken together, although RFA remains the most established technique, MWA represents an emerging and promising option, particularly in cases where rapid and uniform energy delivery is advantageous [4].

From a technical perspective, RFA generates resistive heating through electrical current and is influenced by tissue impedance, requiring precise positioning of the electrode within the nidus for complete ablation [2]. In contrast, MWA produces higher temperatures in a shorter time, distributes heat more homogeneously, and is less affected by tissue composition or impedance [1,5]. These differences suggest that MWA may be advantageous in sclerotic bone or lesions with irregular morphology; however, its safety profile must be carefully considered due to the risk of unintended thermal spread [6]. Although the evidence supporting MWA remains limited to smaller series and recent systematic reviews, these studies generally report comparable efficacy, while emphasizing the need for larger, controlled investigations [1,5,6]. Therefore, the relative merits of RFA and MWA remain incompletely defined, underscoring the need for direct comparative analyses, such as the present study.

Despite widespread adoption of percutaneous ablation for symptomatic OO, the optimal modality remains uncertain because the evidence base is unbalanced: RFA is supported by large series and reviews with consistently high clinical success but also heterogeneous protocols and outcome definitions [4,7]. By contrast, MWA offers biophysical advantages (faster, higher, and more homogeneous heating with less sensitivity to impedance) and encouraging results [8]. However, the literature consists mainly of small, single-center cohorts and a recent systematic review that highlights marked heterogeneity and the absence of randomized or adequately powered head-to-head comparisons [4,5,6]. Few direct comparative studies have been conducted; when performed, long-term efficacy appears similar between RFA and MWA, but small sample sizes limit inferences on recurrence and safety differentials [8].

In addition, lesion- and technique-related prognostic factors (e.g., nidus size and location, proximity to critical structures, and energy/time delivery) are inconsistently analyzed across reports, which impedes modality-specific patient selection [4,7].

To our knowledge, this study represents the largest retrospective series directly comparing RFA and MWA for the treatment of OO, with well-balanced patient groups and extended follow-up. By systematically analyzing efficacy, recurrence, safety, and lesion- or technique-related prognostic factors, it aims to provide a more robust evidence base than prior single-center or small-scale reports. Beyond clarifying the relative performance of these two modalities, our findings are expected to inform patient selection, optimize procedural decision-making in daily practice, and guide the design of future prospective and randomized investigations.

## 2. Materials and Methods

### 2.1. Study Design and Setting

This retrospective study was conducted at the Başkent University Adana Dr. Turgut Noyan Application and Research Center, Department of Interventional Radiology, between January 2014 and January 2023. It included patients who underwent percutaneous RFA or MWA for OO treatment during this period. The protocol was reviewed and approved by the Başkent University Institutional Review Board (Project no: KA24/327), and all procedures were performed in accordance with the Declaration of Helsinki.

### 2.2. Patient Selection

All patients who underwent CT-guided percutaneous ablation for OO between January 2014 and January 2023 were retrospectively reviewed. The diagnosis of OO was based on a combination of clinical presentation (typical nocturnal pain responsive to NSAIDs), radiological findings (characteristic nidus on CT and/or MRI), and multidisciplinary consensus involving radiologists and orthopedic surgeons.

#### 2.2.1. Inclusion Criteria

1. Radiologically and clinically confirmed OO, with typical clinical presentation and characteristic imaging features documented in the record.
2. Treatment at our institution with CT-guided percutaneous RFA or MWA as the index ablation, undertaken as either of the following:
  ○ Primary therapy;
  ○ Salvage index ablation after unsuccessful prior treatment (e.g., surgery or percutaneous ablation performed outside our institution or before the study window), provided that (i) a viable/residual nidus was demonstrated on MRI and (ii) failure was adjudicated by our multidisciplinary tumor board (interventional radiology and orthopedics).
3. Complete procedural and clinical documentation, including available pre- and post-procedural VAS pain scores, and a minimum 24-month follow-up after the index ablation.

#### 2.2.2. Exclusion Criteria

1. Incomplete clinical/procedural data or missing pre- or post-procedural VAS scores.
2. Follow-up <24 months after the index ablation.
3. Atypical or discordant imaging/clinical findings that preclude a confident diagnosis of osteoid osteoma.
4. Retreatments (repeat percutaneous ablations) performed at our institution after the index ablation during the study window for early failure or recurrence—not counted as new cases; when present, they were analyzed only as outcomes of the index case, not as separate participants.

At our institution, radiofrequency ablation was performed until microwave ablation equipment became available. Following the acquisition of the MW system, all subsequent cases were treated with microwave ablation.

### 2.3. Procedural Technique

All ablations were performed by two interventional radiologists (I.K. and M.M.), each with over five years of experience in percutaneous tumor ablation. All procedures followed an identical CT-guided technique and standardized protocol under sterile conditions and anesthesiology-supervised intravenous sedation with local anesthesia at the puncture site. Patients were positioned prone or supine according to lesion location, and vital signs were continuously monitored throughout the procedure.

All procedures were performed under CT guidance using a Siemens SOMATOM Emotion 16 scanner (Forchheim, Germany). Then, 1 mm planning CT and CT fluoroscopy were used for real-time trajectory control. A coaxial bone access needle was advanced into the nidus, followed by the insertion of the ablation electrode or antenna. The sheath was withdrawn before energy delivery.

**Microwave ablation (MWA):** Performed using the Canyon KY-2000A Microwave Ablation Generator with an integrated cooling system (Canyon Medical Inc., Nanjing, China). Sixteen-gauge antennas with 3 mm or 7 mm active tips (15 cm shaft) were selected according to nidus size and proximity to critical structures. Ablation was performed at 40–50 W for 2–2.5 min per cycle, with continuous internal cooling to minimize thermal spread.

**Radiofrequency ablation (RFA):** Conducted using the Covidien Cool-tip™ RF Ablation System (Medtronic, Minneapolis, MN, USA). Seventeen-gauge electrodes (RFA1507 and RFA1510; 7 or 10 mm active tips) were used in temperature-controlled mode at 90 °C for 5–6 min, with automatic power modulation to maintain the set temperature.

Three orthogonal nidus diameters were measured on pre-procedural CT multiplanar reformats. The eccentricity index (EI) was calculated as the ratio of the largest to the smallest orthogonal diameter, providing a quantitative measure of nidus shape on CT. A higher EI indicates a more elongated or irregular geometry, which may affect heat distribution and ablation completeness. This definition and rationale were adapted from previous CT-based RFA studies that identified geometric factors, such as nidus size and EI, as potential predictors of treatment outcome [9,10].

### 2.4. Post-Procedural Care

Immediately after ablation, a limited CT scan was obtained to confirm complete nidus coverage and rule out complications. Hemostasis was achieved via manual compression, and the puncture site was covered with a sterile dressing. Patients were monitored briefly, discharged the same day with standard post-procedure instructions and analgesics as needed, and scheduled for early outpatient follow-up within 1–2 weeks.

### 2.5. Follow-Up Protocol and Outcome Definitions

#### Follow-Up Schedule

Within 24–48 h, patients were contacted by phone for pain assessment using the visual analog scale (VAS, 0–10) and to document analgesic use. Early complications, such as skin burn, fever, or neurological symptoms, were screened during this follow-up. Rapid pain reduction was expected following thermal ablation.

At 1–2 weeks, patients were re-evaluated at the outpatient clinic through physical examination, repeat VAS assessment, and the documentation of analgesic cessation and return to normal activity. At 1 month, the early treatment response was determined; MRI was performed in cases of persistent pain (VAS ≥ 3) or atypical symptoms to exclude residual nidus. Further clinical evaluations were scheduled at 3, 6, 12, and 24 months to assess VAS scores, function, and possible recurrence. Routine imaging was reserved for symptomatic patients, as a symptom-based surveillance strategy was preferred [11].

Patients with cortical or diaphyseal lesions resumed partial weight-bearing after 2–3 weeks and progressed as tolerated, whereas juxta-articular or subarticular lesions in weight-bearing bones required restricted mobilization for 4–6 weeks. Non-opioid analgesics were prescribed as needed, with early discontinuation of NSAIDs expected in successfully treated cases.

### 2.6. Outcome Definitions

#### 2.6.1. Technical Success

Technical success was defined as successful coaxial access and intranidal placement of the ablation electrode or antenna, delivery of the planned ablation cycle(s) without premature termination, and session completion without the need for additional unplanned access.

#### 2.6.2. Early Response (24–48 H and 1–2 Weeks)

Early treatment response was defined as a reduction in typical nocturnal pain, quantified based on the VAS, with decreased or discontinued use of analgesics. Both absolute VAS scores and changes in VAS (ΔVAS) were documented.

#### 2.6.3. Clinical Success

Clinical success was evaluated at both early (<4 months) and long-term (>12 months) follow-up:

- **Early clinical success** was defined as the resolution or near resolution of typical pain (VAS ≤ 1) without the need for regular analgesics.
- **Long-term clinical success** was defined as the continuation of these criteria beyond 12 months.
- Recurrence, defined as the reappearance of typical pain after an initial pain-free period, was considered a failure in long-term clinical success analysis.

Clinical failure was defined as not meeting the above criteria.

### 2.7. Recurrence

Recurrence was defined as the reappearance of typical pain at the index site following an initial pain-free interval. In suspected cases, confirmatory imaging with MRI (or thin-slice CT when necessary) was performed.

### 2.8. Time-to-Event Endpoints

- Time to pain relief: Interval from ablation to the first documentation of VAS ≤ 1 without regular analgesic use.
- Time to recurrence: Interval from ablation to the return of typical pain fulfilling the recurrence definition.

### 2.9. Complications

All adverse events were recorded prospectively and graded according to the Society of Interventional Radiology (SIR) classification system (A–F, minor vs. major) [12].

- **Minor complications (SIR A/B)** included self-limited post-procedure pain, transient neuropraxia, small hematoma, or superficial skin injury/infection treated conservatively.
- **Major complications (SIR C–F)** included osteomyelitis or soft-tissue abscess, significant skin burn requiring medical or surgical intervention, insufficiency/subarticular fractures, persistent neurological deficits, hardware breakage requiring retrieval, or events necessitating hospitalization, transfusion, or surgery.

### 2.10. Retreatment Criteria and Imaging Algorithm

In cases where pain persisted or recurred after an initial pain-free interval, targeted MRI was performed to evaluate the ablation zone and exclude residual nidus. Repeat ablation was performed when viable nidus or characteristic imaging findings were identified.

### 2.11. Optional Research MRI

In a predefined subset of patients, particularly those with juxta-articular lesions or for comparative evaluation of ablation zones between RFA and MWA, non-contrast MRI was obtained approximately 4–6 weeks after the procedure. Three orthogonal diameters of the ablation zone were measured. This was performed for research purposes only and was not part of routine follow-up care.

### 2.12. Statistical Analysis

All data were analyzed using R software (version 4.1.1). The normality of the distribution of continuous variables was assessed using the Shapiro–Wilk test. Quantitative variables are expressed as mean ± standard deviation and median (Q1–Q3), while categorical variables are presented as frequencies and percentages.

Associations between categorical variables were examined using Fisher’s exact test and the Monte Carlo-corrected Fisher’s exact test when appropriate. Continuous variables that were not normally distributed between two independent groups were compared using the Mann–Whitney U test. Comparisons of paired, non-normally distributed measurements within groups at two time points were evaluated with the Wilcoxon signed-rank test.

The effect of independent variables on the likelihood of clinical failure was examined using binary logistic regression analysis. The impact of independent variables on recurrence-free survival was assessed using Cox regression analysis. In all statistical tests, a two-tailed *p*-value < 0.05 was considered statistically significant. Given the modest sample size (n = 53), regression analyses should be interpreted with caution and considered exploratory.

## 3. Results

A total of 53 patients (38 males [71.7%], 15 females [28.3%]) were included, with a median age of 17 years (range, 7–46 years). Lesions were most frequently located in the femoral neck, mid- and proximal femoral diaphysis, and were right-sided in 33 patients (62.3%). Most lesions were cortical (42 patients, 79.3%) and without prior treatment (50 patients, 94.3%). In total, 26 patients (49.1%) underwent MWA and 27 (50.9%) underwent RFA (Table 1).

### 3.1. Technical and Clinical Outcomes

Technical success was achieved in all cases (53/53, 100%). Early clinical success was achieved in 50 patients (94.3%), and long-term clinical success (≥12 months) was maintained in 48 patients (90.6%). Three patients (5.7%) required additional analgesics post-procedure. The median follow-up duration was 3 years (IQR 2–4). Pain scores improved markedly from a median VAS of 10 to 0 (*p* < 0.001), with a median ΔVAS of 10 and a median time to pain relief of 2 days (Table 1).

### 3.2. Predictors of Clinical Success and Recurrence

Clinical success did not differ significantly between the MWA (25/26 [96.2%]) and RFA (25/27 [92.6%]) groups (*p* = 1.000). Lesions ≥ 10 mm showed lower success (9/12 [75.0%]) than those <10 mm (41/41 [100%], *p* = 0.009). Recurrence occurred in two patients overall (4%), one per treatment group, with no significant association to ablation type (*p* = 1.000) or nidus size (*p* = 0.331). The eccentricity index (EI) was not significantly associated with clinical success (*p* = 0.238) or recurrence (*p* = 1.000) (Table 2).

### 3.3. Regression Analyses

Binary logistic regression identified nidus diameter as a significant predictor of clinical failure in univariate analysis (OR, 1.449; 95% CI, 1.045–2.010; *p* = 0.026), while the eccentricity index showed a borderline association (OR, 2.782; 95% CI, 1.137–6.807; *p* = 0.025). However, none of the parameters remained significant in the multivariate model (Table 3). Cox regression analysis showed no variables with a statistically significant impact on recurrence-free survival (*p* > 0.05) (Table 4).

### 3.4. Procedure Parameters

MWA demonstrated a significantly shorter ablation duration (median of 2 min [IQR 2–2]) compared with RFA (6 min [IQR 5–6]; *p* < 0.001), whereas their total procedure times were similar (28.5 vs. 31 min, *p* = 0.215). There were no significant differences in time to pain relief, complete response, or return to activity between groups (all *p* > 0.05). Follow-up duration was longer in the RFA group (median of 4 years vs. 3 years, *p* < 0.001) (Table 5).

### 3.5. Pain Scores

The median pre-procedural VAS score was 10 in both the MW and RF groups, with no significant difference between the devices (*p* = 0.103). Post-procedural median VAS was 0 in both groups (*p* = 0.739), and the median ΔVAS was 10, with no between-group difference (*p* = 0.165). Within each group, VAS decreased significantly from 10 pre-procedure to 0 post-procedure (MW: *p* < 0.001; RF: *p* < 0.001) (Table 6).

### 3.6. Nidus Morphology and Complications

**Figure 1:**
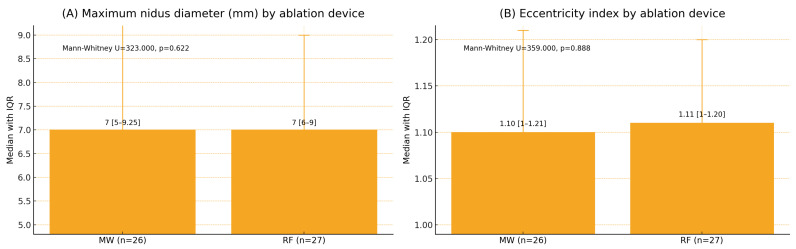
Maximum nidus diameter and eccentricity index by ablation device (median [Q1–Q3]). Note: MW: microwave ablation; RF: radiofrequency ablation; Q1–Q3: interquartile range. x Mann–Whitney U test. Values are presented as median (Q1–Q3). Panel (**A**): U = 323.000, *p* = 0.622; Panel (**B**): U = 359.000, *p* = 0.888. Two-sided *p* < 0.05 was considered statistically significant.

**Figure 2:**
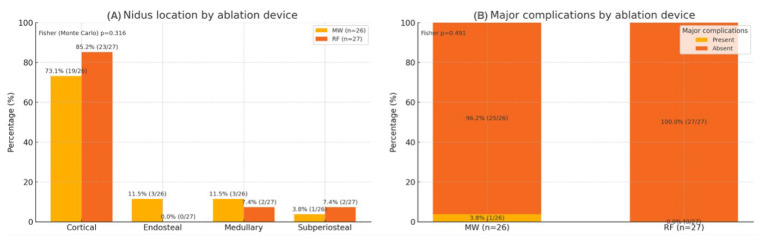
Distribution of nidus location and occurrence of major complications according to ablation device. MW: microwave ablation; RF: radiofrequency ablation. Values show the percentage within each device group, labeled as % (n/N). Fisher (Monte Carlo) *p* = 0.316 for nidus location; Fisher *p* = 0.491 for major complications. Two-sided *p* < 0.05 is considered statistically significant.

The median nidus diameter was 7 mm in both groups (*p* = 0.622), and the median EI was 1.10 for MWA and 1.11 for RFA (*p* = 0.888) (Figure 1). The nidus location distribution was similar between modalities (*p* = 0.316). One major complication (third-degree skin burn) occurred after the MWA of a calcaneal lesion (1/53, 1.9%), while no minor complications were reported (Figure 2).

Key parameters that influenced treatment outcomes—including nidus size, lesion geometry, and procedural differences between RFA and MWA—are summarized in Table 7.

## 4. Discussion

In this retrospective series, both RFA and MWA achieved high technical and clinical success rates in the management of OO, with low complication and recurrence rates. These outcomes are consistent with previous reports describing percutaneous thermal ablation as a reliable first-line treatment for OO [4,6,7]. Importantly, our study represents the largest single-center cohort directly comparing RFA and MWA with extended follow-up. Nevertheless, the interpretation of our results requires caution, given several methodological and contextual considerations.

Our results (100% technical success, 94.3% clinical success, 4% recurrence, and 1.9% major complications) were comparable to previously published data, which consistently report >90% clinical success and 2–3% complication rates for both RFA and MWA [1,4,5]. The 96.2% success rate observed in our MWA group aligns with these findings, supporting similar efficacy between modalities. Although some studies suggested slightly higher technical success for MWA [8], these were limited by small cohorts and heterogeneous follow-up. Overall, our data indicate that treatment outcomes depend more on lesion characteristics than on the ablation device itself.

In our cohort, the 4% recurrence rate was comparable with prior reports [13]. Similarly to most published series, recurrence was defined and monitored primarily based on clinical symptoms, reflecting the pragmatic reality that routine imaging in asymptomatic patients may overestimate incomplete ablation and expose patients to unnecessary radiation [14,15]. Nonetheless, this approach carries the inherent limitation of potentially missing subclinical or asymptomatic recurrences, as highlighted in the literature [10,13]. Our protocol, therefore, prioritized symptom-based follow-up, with targeted CT or MRI reserved for cases of persistent or recurrent pain. While this strategy is practical and consistent with clinical standards, it underscores the need for cautious interpretation of recurrence rates and for further studies incorporating systematic imaging to clarify the true burden of residual disease.

The only major complication in our series was a third-degree skin burn that occurred following the MW ablation of calcaneal osteoid osteoma (1.9%). This event was considered an anticipated risk due to the thin soft-tissue coverage of the calcaneus and the short skin–nidus distance. As highlighted in the literature, the deeper tissue penetration of MW energy increases the risk of thermal injury, particularly in subcutaneous bones [16]. To mitigate this risk, several protective strategies have been recommended, including shortening ablation time, using smaller active-tip probes, applying cold compresses over the skin, and performing subcutaneous hydrodissection to increase the skin–nidus distance [14]. Furthermore, careful selection of the ablation modality—such as considering cryoablation for superficial sites—may further reduce the likelihood of severe adverse events [16]. Our case aligns with prior observations that superficial locations carry a higher risk of skin injury, where open surgery may be preferred [17].

Although MWA in our series demonstrated a significantly shorter ablation duration compared with RFA (2 vs. 6 min, *p* < 0.001), this did not translate into a reduction in overall procedure time (28.5 vs. 31 min, *p* = 0.215). This finding aligns with prior reports: meta-analyses have demonstrated markedly shorter ablation times with MWA (≈approximately 1.6 min vs. 6.9 min for RFA), but similar procedure times of around 70 min for both modalities [13]. As highlighted in earlier RFA reviews, the majority of room time is determined by patient preparation, positioning, trajectory planning, and coaxial access rather than the ablation cycle itself [14,15]. Therefore, while MWA provides technical efficiency in energy delivery, its practical impact on workflow efficiency appears limited.

In our series, nidus diameter emerged as the strongest predictor of clinical outcome, with lesions ≥ 10 mm demonstrating a significantly higher likelihood of treatment failure and the need for reintervention (*p* = 0.009). This observation is in line with previous RFA research reporting an increased risk of incomplete ablation and recurrence in lesions ≥ 10 mm [2]. However, it is noteworthy that most published MWA studies—despite reporting high overall success rates—have not systematically evaluated nidus size as an independent prognostic factor. For instance, Parisot et al. [5] reported a long-term clinical success rate of 93% but did not stratify outcomes according to nidus diameter [5]. Similarly, Mutlu et al. [1], in their 59-patient cohort, documented a 96.6% success rate, yet found no correlation between lesion volume or diameter and clinical response [1]. A recent systematic review encompassing 143 MWA cases reported an overall recurrence rate of 2.8%, but again without examining lesion size or geometric parameters [6]. While this topic has been comprehensively discussed in the RFA literature, it has rarely been examined in the context of MWA. Our findings suggest that nidus diameter may likewise have clinical relevance in MWA, emphasizing the need for careful probe positioning, thorough pre-procedural planning, and optimization of energy delivery in lesions ≥10 mm.

Our study also provides a novel perspective regarding the eccentricity index (EI). To date, the EI has been scarcely investigated, and its occurrence has been reported only in selected RFA cohorts. Baal et al. [10] suggested that an EI ≥ 3, along with female sex and younger age (<13 years), may represent risk factors for symptomatic recurrence [10]. Nevertheless, these findings have not been validated across larger or independent series, and importantly, the EI has never been evaluated in MWA cohorts. In our study, the EI reached statistical significance on univariate analysis and remained borderline significant in multivariate modeling, suggesting that lesion geometry may influence heat distribution and ablation completeness. Recent evidence from Tung et al. [9] further supports this concept, demonstrating that an elongated morphology—defined as a nidus length greater than 10 mm combined with an EI of 3 or higher—was significantly associated with RFA treatment failure in children [9]. Collectively, these data highlight geometry as a neglected but clinically relevant factor that may contribute to outcome variability in OO, particularly in MWA-treated lesions. While our data do not directly prove a causal link, they suggest that even minor deviations in probe positioning within the nidus could potentially affect ablation completeness and clinical success, especially in elongated or eccentrically shaped lesions. These findings highlight the importance of careful pre-procedural planning and optimizing probe placement according to nidus morphology. Multiplanar CT assessment enables accurate evaluation of nidus geometry and orientation, helping to determine the optimal trajectory and active-tip position. In elongated or eccentrically shaped nidi, individualized planning with overlapping or multi-cycle ablation may improve completeness and long-term clinical success.

In this single-center, head-to-head series, RFA and MWA yielded comparable technical and clinical success rates, with low recurrence. The only clear advantage of MWA was a shorter ablation time, which did not translate into a shorter overall procedure duration. These findings indicate that treatment selection should prioritize lesion characteristics rather than device type: a nidus diameter ≥ 10 mm carried a higher risk of failure, while the eccentricity index showed a borderline association. For superficial bones, the possibility of thermal spread warrants protective maneuvers (e.g., hydrodissection, shorter/lower-energy cycles) and, in select cases, consideration of alternative modalities. Interpretation of the findings is limited by the retrospective, single-center design and the imbalance in follow-up duration between groups. Although no formal matching or propensity scoring was applied between the RFA and MWA cohorts, baseline demographic and lesion characteristics (age, sex, lesion location, nidus size, and morphology) were compared to ensure overall similarity. All ablations were performed by experienced interventional radiologists using a standardized CT-guided technique; thus, operator variation was minimal. Nevertheless, the involvement of multiple operators may still limit generalizability, and multicenter prospective studies with standardized protocols are warranted to validate these results and to further clarify the prognostic influence of lesion morphology and size across ablation modalities.

## 5. Conclusions

RFA and MWA provide high and similar effectiveness for symptomatic osteoid osteoma; although MWA shortens ablation time, it does not meaningfully reduce total room time. Therapy should be individualized to lesion-specific factors—especially nidus size ≥10 mm and lesion geometry—rather than the energy platform itself. These results suggest that treatment planning in osteoid osteoma should be individualized according to nidus morphology rather than energy modality, supporting a lesion-oriented rather than device-oriented approach.

## Figures and Tables

**Table 1:** **Descriptive statistics of the study** **population.**

	Frequency	Percentage (%)
Gender		
Male	38	71.7
Female	15	28.3
Localization of the lesion		
Femur neck	11	20.8
Proximal tibial diaphysis	7	13.3
Distal femoral diaphysis	5	9.4
Proximal femoral diaphysis	9	17
Mid-femoral diaphysis	10	19
Proximal humeral diaphysis	1	1.9
Calcaneus	1	1.9
Mid-tibial diaphysis	1	1.9
Greater trochanter of the femur	1	1.9
Iliac bone	1	1.9
Pedicle of the L5 vertebra	1	1.9
Talus	1	1.9
Humeral head	1	1.9
Neck of the scapular spine	1	1.9
Mid-humeral diaphysis	1	1.9
Distal tibial diaphysis	1	1.9
Laterality		
Right	33	62.3
Left	20	37.7
History of prior treatment		
None	50	94.3
Open surgery	1	1.9
MW ablation	1	1,9
RF ablation	1	1.9
Ablation device		
MW	26	49.1
RF	27	50.9
Nidus location		
Cortical	42	79.3
Endosteal	3	5.7
Medullary	5	9.4
Subperiosteal	3	5.7
Technical success		
Yes	53	100
Early Clinical success (≤4 months)		
Yes	50	94.3
No Long-term clinical success (≥12 months) Yes No	3 48 5	5.7 90.6 9.4
Ablation energy parameters		
40 W	11	20.8
50 W	15	28.3
90 °C (target temperature)	27	50.9
Presence of recurrence		
Yes	2	4
No	48	96
Minor complications		
No	53	100
Major complications		
Yes	1	1.9
No	52	98.1
Post-procedural additional analgesic requirement		
Yes	3	5.7
	Mean ± SD	Median (Q1–Q3)
Age	18.98 ± 7.95	17 (7–46)
Ablation duration (minutes)	3.85 ± 1.8	5 (2–6)
Pre-procedural VAS score	9.77 ± 0.47	10 (10–10)
Post-procedural VAS score	0.38 ± 1.26	0 (0–0)
Change in VAS score (ΔVAS)	9.4 ± 1.35	10 (9–10)
Time to pain response (days)	2.06 ± 0.91	2 (1–2.75)
Time to complete response (days)	5.38 ± 2.99	4,5 (3–6)
Time to recurrence (months)	9 ± 2.83	9 (8–10)
Time to return to activity (days)	5.18 ± 3.41	4 (3–5)
Follow-up duration (years)	3.22 ± 1.41	3 (2–4)
Symptom duration (months)	9.4 ± 12.97	6 (4–9)
Total procedure time (minutes)	30.36 ± 9.45	30 (25–35)
Nidus diameter (maximum, mm)	7.87 ± 3.28	7 (6–9)
Eccentricity index (EI)	1.15 ± 0.22	1.11 (1–1.2)

Data are presented as frequency (percentage). MW, microwave; RF, radiofrequency; VAS, visual analog scale. Data are presented as mean ± standard deviation (SD) and median (Q1–Q3). VAS, visual analog scale; EI, eccentricity index; ΔVAS, difference between pre- and post-procedural VAS scores.

**Table 2:** Association of device, nidus diameter, and eccentricity index with clinical success and recurrence.

Factor	Category	Clinical Success			Recurrence		
		Successful n (%)	Unsuccessful n (%)	*p* ^x^	Yes n (%)	No n (%)	*p* ^x^
Ablation Device	MW	25 (96.2)	1 (3.9)	1.000	—	—	—
	RF	25 (92.6)	2 (7.4)		—	—	—
Nidus diameter (maximum, mm)	<10 mm	41 (100)	0 (0)	0.009	1 (2.4)	40 (97.6)	0.331
	≥10 mm	9 (75)	3 (25)		1 (11.1)	8 (88.9)	
Eccentricity index	1	25 (100)	0 (0)	0.238	1 (4)	24 (96)	1.000
	≠1	25 (89.3)	3 (10.7)		1 (4)	24 (96)	

Notes: ^x^ Fisher’s Exact test; values are presented as n (%). “—”: parameter not reported in that analysis.

**Table 3:** Binary logistic regression analysis of predictors of clinical failure.

	Clinical Success		Univariate		Multiple	
	Success	Failure	OR (%95 CI)	*p*	OR (%95 CI)	*p*
Ablation device						
MW	25 (96.2)	1 (3.8)	Reference			
RF	25 (92.6)	2 (7.4)	2 (0.17–23.495)	0.581	1.29 (0.011–148.065)	0.916
Nidus diameter (max, mm)	7.56 ± 3.11	13 ± 1	1.449 (1.045–2.01)	0.026	1.244 (0.683–2.265)	0.476
Eccentricity index	11.12 ± 1.46	17.53 ± 3.14	2.782 (1.137–6.807)	0.025	2.441 (0.979–6.083)	0.055

OR (%95 CI): OR, odds ratio; CI, confidence interval. MW: microwave ablation; RF: radiofrequency ablation. For device type, MW was used as the reference category and compared with RF. Continuous variables (nidus diameter, eccentricity index) are presented as mean ± standard deviation (SD). A *p*-value < 0.05 was considered statistically significant.

**Table 4:** Cox regression analysis of the effect of independent variables on recurrence-free survival.

	Recurrence		Univariate		Multiple	
	No	Yes	HR (%95 CI)	*p*	HR (%95 CI)	*p*
Ablation device						
MW	24 (96)	1 (4)	Reference			
RF	24 (96)	1 (4)	1.021 (0.064–16.32)	0.988	1.288 (0.066–25.18)	0.868
Nidus diameter (max, mm)	7.48 ± 3.11	9.5 ± 3.54	1.155 (0.84–1.589)	0.375	1.147 (0.774–1.701)	0.494
Eccentricity index	11.1 ± 1.45	11.65 ± 2.33	1.235 (0.57–2.673)	0.593	1.071 (0.407–2.815)	0.89

HR (%95 Cl): HR, hazard ratio; CI, confidence interval. MW: microwave ablation; RF: radiofrequency ablation. For device type, MW was used as the reference category and compared with RF. Continuous variables (maximum nidus diameter, eccentricity index) are presented as mean ± standard deviation (SD). A *p*-value < 0.05 was considered statistically significant.

**Table 5:** **Comparison of quantitative variables according to the ablation device** **used.**

	Ablation Device		Test Statistic	*p* ^x^
	MW	RF		
Procedure duration (min)	28.5 (21.75–35.25)	31 (25–35)	281.000	0.215
Recovery time (days)	4 (3–5)	4 (3–8.5)	300.500	0.820
Ablation duration (min)	2 (2–2)	6 (5–6)	0.000	<0.001
Time to pain relief (days)	2 (1–2.5)	2 (2–3)	251.000	0.205
Time to complete response (days)	4 (3.5–6)	5 (3–6.5)	307.000	0.921
Follow-up duration (years)	3 (2–3)	4 (3–5)	169.000	<0.001

MW: microwave ablation; RF: radiofrequency ablation; min: minutes; Q1–Q3: interquartile range. ^x^ Mann–Whitney U test; values are presented as median (Q1–Q3). A *p*-value < 0.05 was considered statistically significant.

**Table 6:** **Comparison of VAS scores by ablation** **device.**

	Ablation Device		Test Statistic	*p* ^x^
	MW	RF		
Pre-procedural VAS score	10 (10–10)	10 (9–10)	416.000	0.103
Post-procedural VAS score	0 (0–0)	0 (0–0)	339.500	0.739
ΔVAS score	10 (9.75–10)	10 (9–10)	415.500	0.165
Test statistic	351,000	378,000		
*p* ^y^	<0.001	<0.001		

MW: microwave ablation; RF: radiofrequency ablation; VAS: visual analog scale; ΔVAS: change in VAS score; Q1–Q3: interquartile range. ^x^ Mann–Whitney U test; ^y^ Wilcoxon signed-rank test. Values are presented as median (Q1–Q3). A *p*-value < 0.05 was considered statistically significant.

**Table 7:** Key prognostic and procedural factors associated with clinical success.

Factor	Category/Threshold	Association with Outcome	*p*-Value
Nidus diameter	≥10 mm	Significantly associated with lower clinical success	0.009
Eccentricity Index (EI)	>1.2	Borderline association with lower success	0.055
Ablation device	MWA vs. RFA	No significant difference	1.000
Ablation duration	—	Shorter in MWA group	<0.001
Follow-up duration	—	Longer in RFA group	<0.001

EI: Eccentricity Index; MWA: Microwave Ablation; RFA: Radiofrequency Ablation.

## Data Availability

The data presented in this study are available on request from the corresponding author. The data are not publicly available due to privacy and ethical restrictions.
